# Deep learning-based prediction of osseointegration for dental implant using plain radiography

**DOI:** 10.1186/s12903-023-02921-3

**Published:** 2023-04-08

**Authors:** Seok Oh, Young Jae Kim, Jeseong Kim, Joon Hyeok Jung, Hun Jun Lim, Bong Chul Kim, Kwang Gi Kim

**Affiliations:** 1 Gil Medical Center, Department of Biomedical Engineering, Gachon University College of Medicine, Incheon, 21565 Korea; 2 Department of Oral and Maxillofacial Surgery, Daejeon Dental Hospital, Wonkwang University College of Dentistry, Daejeon, 35233 Korea

**Keywords:** Dental Digital radiography, Deep learning, Artificial Intelligence, Dental Implant, Osseointegration, Oral Surgical Procedures

## Abstract

**Background:**

In this study, we investigated whether deep learning-based prediction of osseointegration of dental implants using plain radiography is possible.

**Methods:**

Panoramic and periapical radiographs of 580 patients (1,206 dental implants) were used to train and test a deep learning model. Group 1 (338 patients, 591 dental implants) included implants that were radiographed immediately after implant placement, that is, when osseointegration had not yet occurred. Group 2 (242 patients, 615 dental implants) included implants radiographed after confirming successful osseointegration. A dataset was extracted using random sampling and was composed of training, validation, and test sets. For osseointegration prediction, we employed seven different deep learning models. Each deep-learning model was built by performing the experiment 10 times. For each experiment, the dataset was randomly separated in a 60:20:20 ratio. For model evaluation, the specificity, sensitivity, accuracy, and AUROC (Area under the receiver operating characteristic curve) of the models was calculated.

**Results:**

The mean specificity, sensitivity, and accuracy of the deep learning models were 0.780–0.857, 0.811–0.833, and 0.799–0.836, respectively. Furthermore, the mean AUROC values ranged from to 0.890–0.922. The best model yields an accuracy of 0.896, and the worst model yields an accuracy of 0.702.

**Conclusion:**

This study found that osseointegration of dental implants can be predicted to some extent through deep learning using plain radiography. This is expected to complement the evaluation methods of dental implant osseointegration that are currently widely used.

**Supplementary Information:**

The online version contains supplementary material available at 10.1186/s12903-023-02921-3.

## Background

Dental implants are widely used for the rehabilitation of edentulous spaces, and osseointegration is essential for the success of dental implants. Various methods have been used to evaluate osseointegration [1–3]. However, the most widely used methods have the disadvantage of being invasive.

Recently, deep learning has been actively applied to dentistry and maxillofacial surgery. The prediction of extraction difficulty and postoperative paresthesia in relation to the mandibular third molar has been described [4, 5]. Studies related to dentofacial dysmorphosis have also been reported [6]. This modality can also help predict the need [7, 8] and outcomes [9] of orthognathic surgery.

Prediction of implant osseointegration through plain radiography and based on deep learning is a potential noninvasive modality; however, it has not yet been studied. Therefore, in this study, we investigated whether plain radiography can help predict osseointegration of dental implants.

## Methods

### Datasets

In this study, panoramic and periapical radiographs of 580 patients (311 men, 269 women; age range, 21–78 years) with 1,206 dental implants, who visited the Daejeon Dental Hospital, Wonkwang University, between January 2015 and December 2018 for dental implant treatment, were used for the training and testing of a deep learning model. In this study, only dental implants placed by a single surgeon after confirming adequate bone healing 3 or more months after tooth extraction were included. Periotest was used to evaluate implant osseointegration 3 or more months after implant placement. All dental implants used in this study satisfied the following conditions: (1) no tenderness on palpation, percussion, or function; (2) no horizontal and/or vertical mobility; (3) no uncontrolled progressive bone loss; (4) no uncontrolled exudate; and (5) no alveolar bone loss around the implant. Implants for which additional bone grafting, including sinus elevation, was performed were excluded. The reverse torque test was performed to confirm successful osseointegration at the time of abutment connection. In all cases, it was confirmed that there was no such problem by observing a lapse of three years or more.

Panoramic and/or periapical radiography were performed immediately after implant placement and after successful osseointegration. Panoramic views were obtained using Promax (Planmeca, Helsinki, Finland; current, 12 mA; voltage, 72 kV; exposure time, 15.8 s) or PCH-2500 (Vatech, Hwaseong, Korea; current, 10 mA; voltage, 72 kV; exposure time, 13.5 s). Planmeca ProX (Planmeca, Helsinki, Finland) was used for periapical radiography.

Group 1 (338 patients, 591 dental implants) included patients who underwent radiography immediately after implant placement, that is, when osseointegration had not yet occurred. Group 2 (242 patients, 615 dental implants) included patients who underwent radiography after confirming successful osseointegration.

### Preprocessing

In this study, the data-cleaning process was not performed. Therefore, deep learning models were trained by raw data. The validation and test processes were also conducted by using raw data.

For segmentation, a semi-automatic process, automatic segmentation based on Otsu’s method [10], was initially used for each dental implant. After the initial segmentation, additional manual correction was performed to clearly define the region of interest (ROI).

**Fig. 1:**
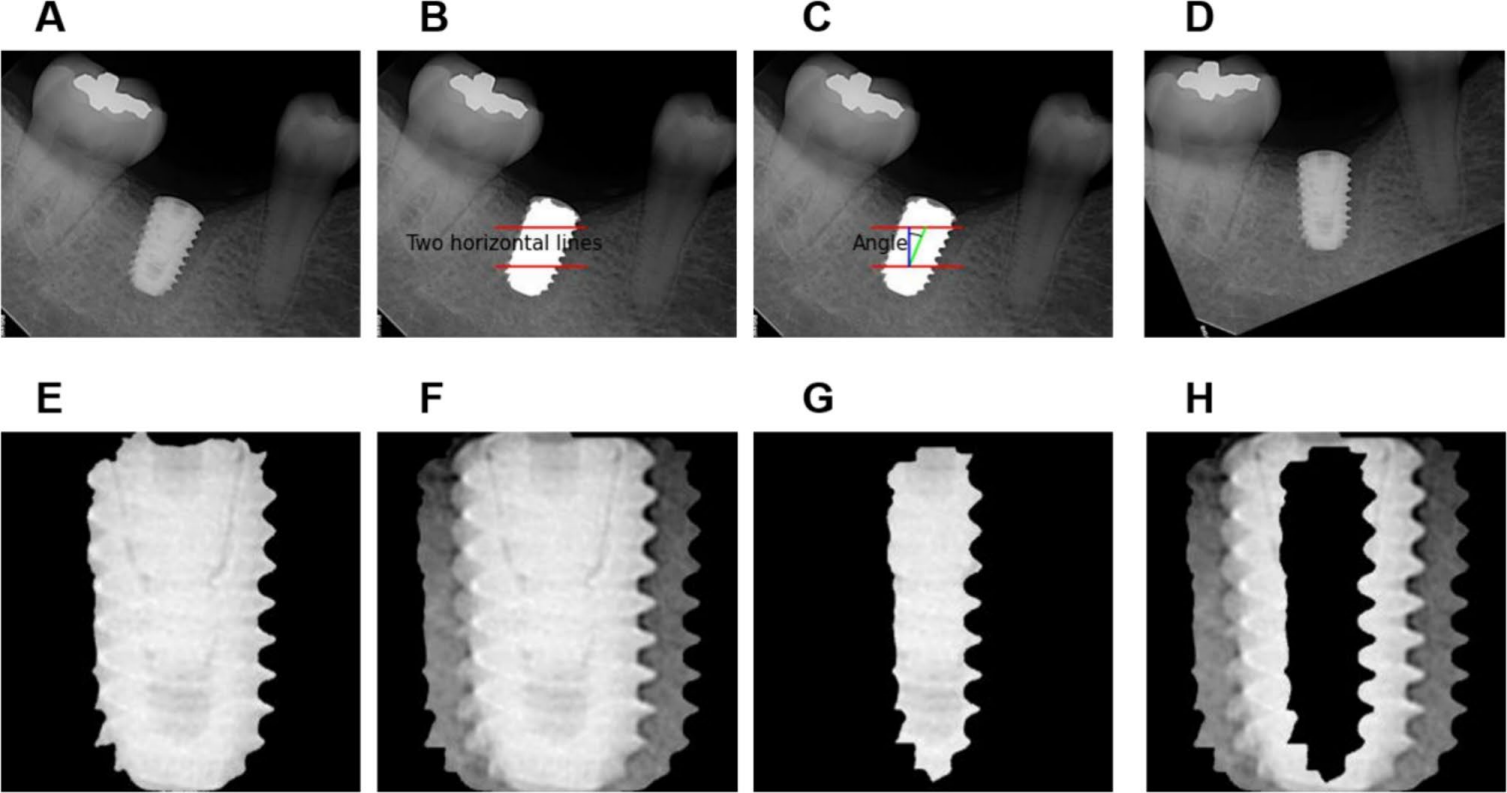
Illustration of the preprocessing step. (A) Raw image; (B) Two horizontal lines on the inner 3/10 and 7/10 portions of the implant region of interest (ROI); (C) Angle calculation for implant ROI rotation; (D) Rotated implant ROI; (E) Cropped image with the rotated implant ROI; (F) Morphological erosion for inner-implant ROI; (G) Morphological dilation for outer-implant ROI; (H) Peri-implant ROI creation by subtracting the inner-implant ROI from the outer-implant ROI

The implant ROI was then aligned in the vertical direction. In this step, two horizontal lines were drawn on the inner 3/10 and 7/10 portions of the implant ROI. The median point of each horizontal line was measured. The angle between the connecting lines of the two points and the vertical line of the image was calculated. The implant ROI was then rotated by this angle to straighten it. This step was required to extract the peri-implant ROI, which was the right and left periphery of the dental implant. The straightening method is shown in Fig. 1A-D.

In the osseointegration prediction, the adjacent bone of the dental implant was an important area, but not the dental implant of itself. Therefore, the implant ROI was converted into a peri-implant ROI to focus on the contact surface between the dental implant and its adjacent bone. Only the right and left peripheries of the dental implant were extracted. The peri-implant ROI was extracted in two sub-steps: (1) the inner-implant ROI was defined by performing morphological erosion at the inner half with a horizontal kernel from the implant ROI. Equally, the outer implant ROI was the morphological dilation into the outer half. (2) The peri-implant ROI was defined by subtracting the inner implant ROI from the outer implant ROI. The peri-implant ROI was used to predict osseointegration in this study and is shown in Fig. 1E-H. Each peri-implant ROI was cropped to the images. Finally, all cropped peri-implant images were zero-padded to a square shape and resized to 256 × 256.

### Model implementation

For osseointegration prediction, we employed seven deep learning models: ResNet-18,34,50 [11], DenseNet-121,201 [12], MobileNet-V2 [13], and MobileNet-V3 [14]. ResNet is a convolutional neural network that uses skip connection to solve the gradient vanishing problem. Skip connection is identity mapping that adds feature maps of a preceding layer into its own feature maps [11]. DenseNet is a convolutional neural network in which all layers are directly connected to each other through a dense block. Therefore, in each layer, the feature maps from all preceding layers are input. This architecture has the advantages of resolving gradient vanishing, reusing features, strengthening feature propagation, and reducing the number of parameters [12]. MobileNet-V2 is a lightweight convolutional neural network that targets mobile and embedded applications. This model is based on an inverted residual block, which is the inverted structure of a conventional residual block. Within the intermediate expansion layer, depth-wise convolution was used to filter the feature maps [13]. MobileNet-V3 is an advanced version of MobileNet-V2 that leverages AutoML techniques [14]. There are two types of models in MobileNet-V3: Small and large. The small MobileNet-V3 model was used in this study.

The cross-entropy loss was used as a loss function. The initial learning rate was set to 0.0001, and the batch size was set to 32. The learning rate was divided by two when the error plateaued during 10 epochs. For model optimization, Adam [15] was employed, with a weight decay of 0.005. The training process was stopped early when the error plateaued during 30 epochs. To avoid overfitting, on-the-fly data augmentation strategies were applied in the training process by random rotation [-45°, 45°], random flip with a probability of 0.5, random scaling [0.8, 1.2], and Gaussian blur with the 3 × 3 kernel and sigma [0.1, 0.15].

**Fig. 2:**
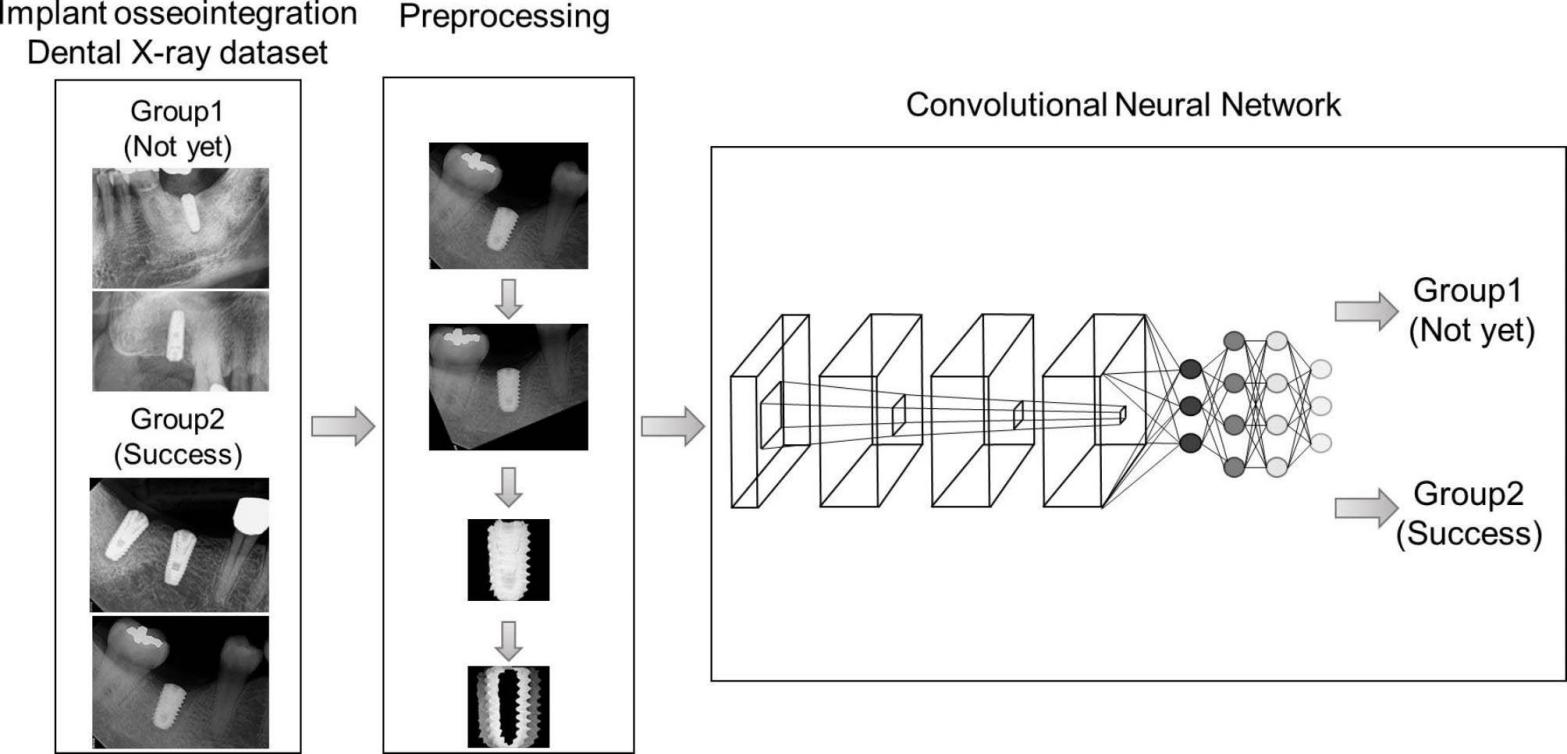
Flowchart for osseointegration prediction. Group 1: radiography was performed immediately after implant placement, that is, when osseointegration had not yet occurred. Group 2: radiography was performed after confirming that osseointegration was successful

Deep learning models were built by performing the experiments 10 times. For each experiment, the dataset was randomly separated into a 60:20:20 ratio of patients for training, validation, and test sets (347, 116, and 117 patients, respectively). A flowchart of the study is presented in Fig. 2.

### Statistical analysis

In this study, Group 1 was set as the negative case, and Group 2 was set as the positive case. The binary prediction for osseointegration was calculated using a threshold of 0.5 on the predicted probability. Consequently, deep learning models were evaluated using four quantitative analysis metrics: specificity, sensitivity, accuracy, area under the receiver operating characteristic curve (AUROC). Specificity was defined as the proportion of images with correct prediction from among all images in Group (1) Sensitivity was defined as the proportion of images with correct prediction from among all images in Group (2) Accuracy was defined as the proportion of correct predictions from among the total number of images. To calculate the AUROC, the receiver operating characteristic (ROC) curve was illustrated by the true and false positive rates across different thresholds for the predicted probability. Thus, the AUROC was defined by the area under the ROC curve.

## Results

**Fig. 3:**
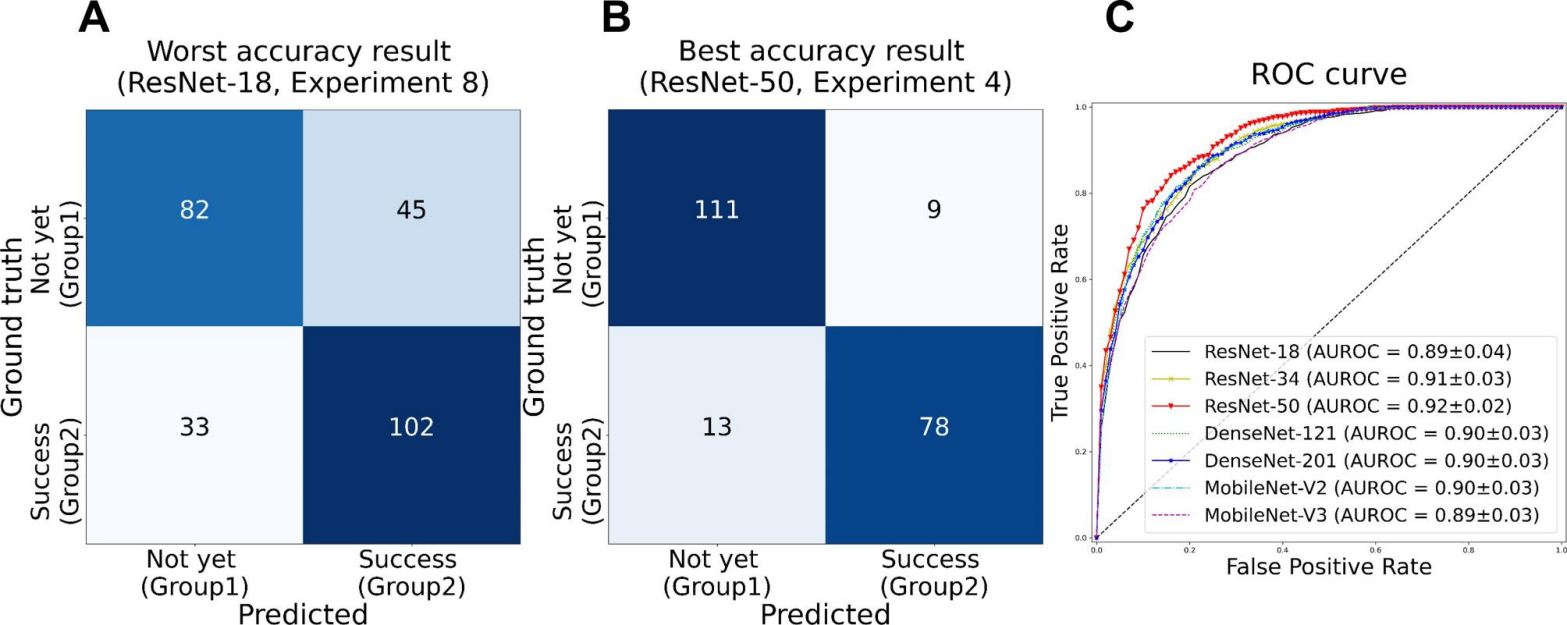
Confusion matrices and receiver operating characteristic (ROC) curves. (A) Confusion matrix of the worst accuracy result; (B) Confusion matrix of the best accuracy result; (C) Average ROC curves of 10 times repeated experiments

**Fig. 4:**
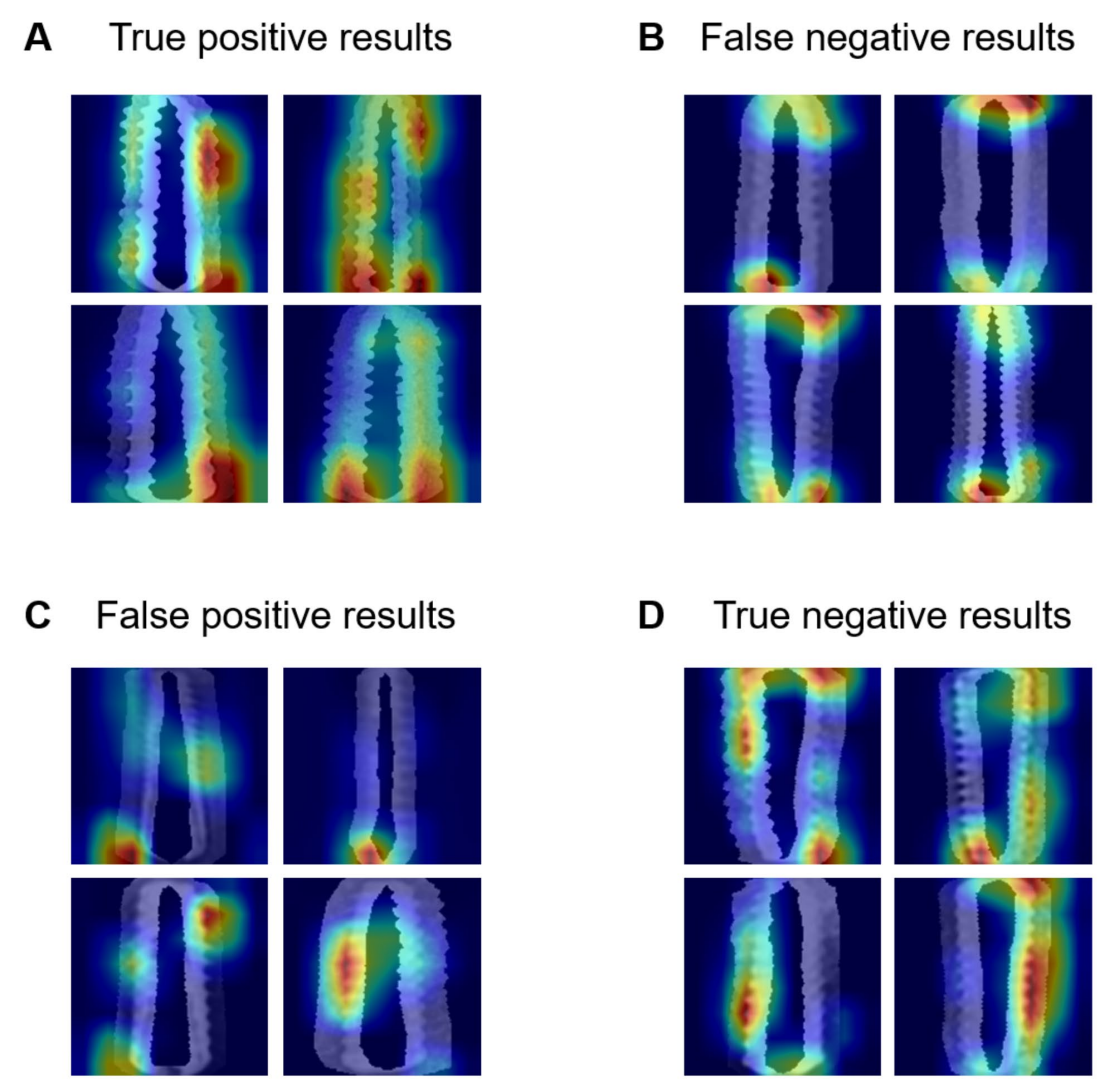
Visualization results of a best accuracy model by Grad-CAM method. (A) True positive results; (B) False negative results; (C) False positive results; (D) True negative results

In this study, seven deep learning models were evaluated using 10 repeated experiments. The classification results of osseointegration are presented in Table 1. The mean values of specificity, sensitivity, and accuracy ranged 0.780–0.857, 0.811–0.833, and 0.799–0.836, respectively. Next, the worst and best accuracies from among the seven deep learning models and 10 experiments were calculated. The lowest accuracy was 0.702 for ResNet-18. In contrast, the highest accuracy was 0.896 for ResNet-50. The confusion matrices for the worst and best accuracy results are shown in Fig. 3A-B. The average ROC curves of the 10 experiments are presented in Fig. 3C. The mean AUROC values ranged from to 0.890–0.922. To understand the decision of interest, Grad-CAM [16] was employed as a visualization method. The red region is where the contribution is high, and the blue region is where the contribution is lower. The visualization results of the best-accuracy model are presented in Fig. 4.

**Table 1:** Specificity, sensitivity, and accuracy of the deep learning models used in this study

	Specificity			Sensitivity			Accuracy		
Model	Mean	Best	Worst	Mean	Best	Worst	Mean	Best	Worst
ResNet18	0.802	0.892	0.646	0.811	0.874	0.690	0.806	0.882	**0.702**
ResNet-34	0.810	0.933	0.687	0.832	0.895	0.748	0.822	0.886	0.759
ResNet-50	0.857	0.925	0.779	0.817	0.903	0.595	0.836	**0.896**	0.734
DenseNet-121	0.823	0.899	0.739	0.813	0.879	0.730	0.818	0.870	0.734
DenseNet-201	0.809	0.915	0.680	0.827	0.942	0.690	0.816	0.854	0.760
MobileNet-V2	0.816	0.883	0.705	0.833	0.922	0.659	0.824	0.883	0.755
MobileNet-V3	0.780	0.883	0.704	0.819	0.890	0.750	0.799	0.853	0.772

## Discussion

Deep learning has been actively applied to dental research. Several deep learning studies related to dental implants have been reported. The marginal bone loss around implants has also been extensively studied [17, 18]. Liu et al. used a balanced database, but the incidence of bone resorption at implant margins was low [17]. And there was a need to improve model performance through standardized radiographs produced via the paralleling technique. Also, the study of Mameno et al. did not evaluate the progression of peri-implantitis and remained unclear [18]. Lerner et al. performed a study on fixed implant prosthodontics [19]. However, their study did not target various implant systems, software and components. Huang et al. attempted to predict the risk of dental implant loss [20]. They studied based on preoperative cone-beam computed tomography and did not target radiography after actual dental implantation. Bayrakdar et al. conducted a study on dental implant planning [21]. They succeeded in the determination of the mandibular canal, but the bone height could not be determined correctly in these regions. Several studies have been conducted to identify and classify dental implants [22, 23]. However, the attention branch network model used by Sukegawa et al. requires a very large amount of calculation cost to obtain the effect size [22]. In the case of Hadj Saïd et al., it is regrettable that a more varied sample was not targeted [23]. To the best of our knowledge, deep learning studies on the osseointegration of dental implants have not yet been reported.

Evaluation of osseointegration for dental implant fixation is important to determine the appropriate timing of placing the superstructure after implantation. Many methods have been proposed to assess the osseointegration, including histomorphological observation of the bone-implant contact interface [1], mobility tests using Periotest [1], removal torque tests to measure rotational removal forces [2], and use of resonance frequencies [3]. Although these methods are still widely used, they have the disadvantage of being invasive. Therefore, we applied deep learning to plain radiography.

In this study, the panoramic and periapical radiographs were combined. To increase the accuracy of the research results, it would be beneficial to further unify the shooting methods. However, although accuracy is important, we did not unify the imaging methods to understand whether the prediction of osseointegration through deep learning is possible in various clinical environments.

Various deep-learning models have been proposed. Therefore, we used seven models in this study and compared their specificity, sensitivity, and accuracy. In addition, because the results can be different each time training, validation, and testing are performed in one model, the test was performed 10 times in each model. As shown in Table 1, osseointegration of dental implants was predicted with an accuracy of approximately 4/5.

## Conclusions

We found that osseointegration of dental implants can be predicted to some extent through deep learning using plain radiography. This is expected to complement the evaluation methods of dental implant osseointegration that are currently widely used.

## Electronic supplementary material

Below is the link to the electronic supplementary material.

Supplementary Material 1

## Data Availability

The datasets generated and analyzed during the current study are not publicly available because the Institutional Review Board of Daejeon Dental Hospital, Wonkwang University, did not allow it, but they are available from the corresponding author upon reasonable request.

## Abbreviations

AUROC: area under the receiver operating characteristic curve
ROI: region of interest
ROC: receiver operating characteristic
