# Supplementary material for: Deep learning-based prediction of osseointegration for dental implant using plain radiography
Source: BMC Oral Health. 2023 Apr 8;23:208. doi: 10.1186/s12903-023-02921-3 (PMC10082489; doi:10.1186/s12903-023-02921-3)
Supplement: Supplementary file 1 — Supplementary Material 1 [file 12903_2023_2921_MOESM1_ESM.docx]

Supplementary material

| Model | 1st | 2nd | 3rd | 4th | 5th | 6th | 7th | 8th | 9th | 10th |
| --- | --- | --- | --- | --- | --- | --- | --- | --- | --- | --- |
| ResNet18 | 0.812 | 0.794 | 0.805 | 0.882 | 0.845 | 0.785 | 0.842 | 0.702 | 0.844 | 0.751 |
| ResNet-34 | 0.825 | 0.867 | 0.846 | 0.886 | 0.795 | 0.826 | 0.806 | 0.767 | 0.844 | 0.759 |
| ResNet-50 | 0.825 | 0.815 | 0.870 | 0.896 | 0.854 | 0.815 | 0.858 | 0.863 | 0.831 | 0.734 |
| DenseNet-121 | 0.870 | 0.802 | 0.833 | 0.834 | 0.824 | 0.815 | 0.846 | 0.786 | 0.836 | 0.734 |
| DenseNet-201 | 0.830 | 0.839 | 0.854 | 0.853 | 0.799 | 0.808 | 0.846 | 0.760 | 0.800 | 0.768 |
| MobileNet-V2 | 0.776 | 0.786 | 0.793 | 0.853 | 0.791 | 0.785 | 0.834 | 0.786 | 0.813 | 0.772 |
| MobileNet-V3 | 0.883 | 0.766 | 0.866 | 0.872 | 0.854 | 0.789 | 0.834 | 0.813 | 0.804 | 0.755 |

Table S1. List of Accuracies of the deep learning models in 10 time experiment.
